# DisV-HPV16, versatile and powerful software to detect HPV in RNA sequencing data

**DOI:** 10.1186/s12879-019-4123-z

**Published:** 2019-05-29

**Authors:** Bingqing Yan, Xinyi Liu, Siwei Zhang, Siyang Yu, Fangjia Tong, Huanhuan Xie, Lianhao Song, Yan Zhang, Lanlan Wei

**Affiliations:** 1 College of Basic Medicine, Harbin Medical University, Harbin, 150081 China; 2 Wu Lien-Ten Institute, Harbin, 150081 China; 3 College of Life Sciences, Harbin Institute of Technology, Harbin, 150081 China

**Keywords:** HPV16 oncogenes, Software, RNA sequencing, Virus

## Abstract

**Background:**

The increasing availability of high-throughput sequencing data provides researchers with unprecedented opportunities to investigate viral genetic elements in host genomes that contribute to virus-linked cancers. Almost all of the available computational tools for secondary analysis of sequencing data detect viral infection or genome integration events. However, viral oncogenes expression is likely of importance in carcinoma. We therefore developed a new software, DisV-HPV16, for the evaluation of HPV16 oncogenes expression.

**Results:**

HPV16 virus and viral oncogenes expression was detected more rapidly using DisV-HPV16 compared to other software. DisV-HPV16 was proved highly convenient for detecting candidate virus after modification of the reference file. The accuracy of DisV-HPV16 was empirically confirmed in laboratory experiments. DisV-HPV16 exhibited greater reliability than other software.

**Conclusions:**

DisV-HPV16 is a new, dependable software to detect virus and viral oncogenes expression through analysis of RNA sequencing data. Use of DisV-HPV16 can yield deeper, more comprehensive insights into virus infection status and viral and host cell gene expression.

## Background

The number of cancer patients has reportedly increased in recent years. It is estimated that there were 14.9 million incident cancer cases and 8.2 million cancer deaths worldwide in 2013 [1]. Approximately 10–15% of human cancers are known to be caused by viruses [2]. Human papillomavirus (HPV) is a sexually transmitted virus causing various benign and malignant diseases including condyloma acuminatum [3], cervical carcinoma(CC) and head and neck squamous carcinoma (HNSC) [4]. Since the first detection by Gissmann in 1982 [5], the presence of human papillomavirus (HPV) in tumor tissue samples from the head and neck, especially oropharyngeal carcinoma, has been increasing worldwide [6]. The HPV family consists of at least 170 different virus types that preferentially infect the mucosa of the genitals [7]. The high-risk HPV type, a sub-group of mucosal HPVs, causes approximately 5% of all human cancers, corresponding to one-third of all virus-induced tumors [8]. Within the high-risk HPV group, HPV16 is the most oncogenic type found in HNSC patients [9].

The detection of viruses in human cancer tissues has significant clinical implications in oncology. Widespread clinical application of next generation sequencing (NGS) and rapid advances in NGS technology in recent years have enhanced the capabilities for virus detection in human cells and enabled large-scale investigations of virus-host interactions [10–12]. Several software tools have been developed including VirusSeq [13], VirusFinder [14] and ViromeScan [15] that apply a computational subtraction algorithm to distinguish viruses within NGS data. VirusSeq and VirusFinder, however, are disadvantaged by extensive time and multiple alignment tools, respectively [16]. Although ViromeScan is less time consuming than either VirusSeq or VirusFinder, it can only be used to determine the taxonomic composition of virome by aligning sequence reads to completely determined viral genomes [15]. Furthermore, nearly all of the computational tools focus on detecting the presence and integration of virus in sequencing data [17], which are considered the key factors in carcinogenesis [18]. From a disease etiology perspective, however, the more important factor may be the expression of viral oncogenes.

HPV16 oncogenes including E5, E6 and E7 are known to contribute to carcinogenesis. E5 negatively regulates the TGF-β signaling pathway [19]. E6 degrades p53 after binding to both p53 and E6-associated protein ligase [20]. E7 binds to pRb and triggers expression of proteins necessary for DNA replication by activating the E2F transcription factor [21]. Neither E6 nor E7 possess intrinsic enzymatic activity, but functions through direct and indirect interactions with host cellular proteins including several well-known tumor suppressors [22].

In light of the increasing incidence of HPV-associated HNSC, the severity of this disease and the roles of HPV E6 and E7 in carcinogenesis, we undertook the development of a new software tool to enable the detection and analysis of HPV16 oncogenes. The resulting DisV-HPV16 software detects HPV16 in RNA sequencing data and determines the expression levels of HPV16 oncogenes. DisV-HPV16 is faster, more sensitive and more accurate than other software tools. Moreover, its reliability was experimentally validated using RT-PCR.

## Implementation

### Data preparation

**Fig. 1:**
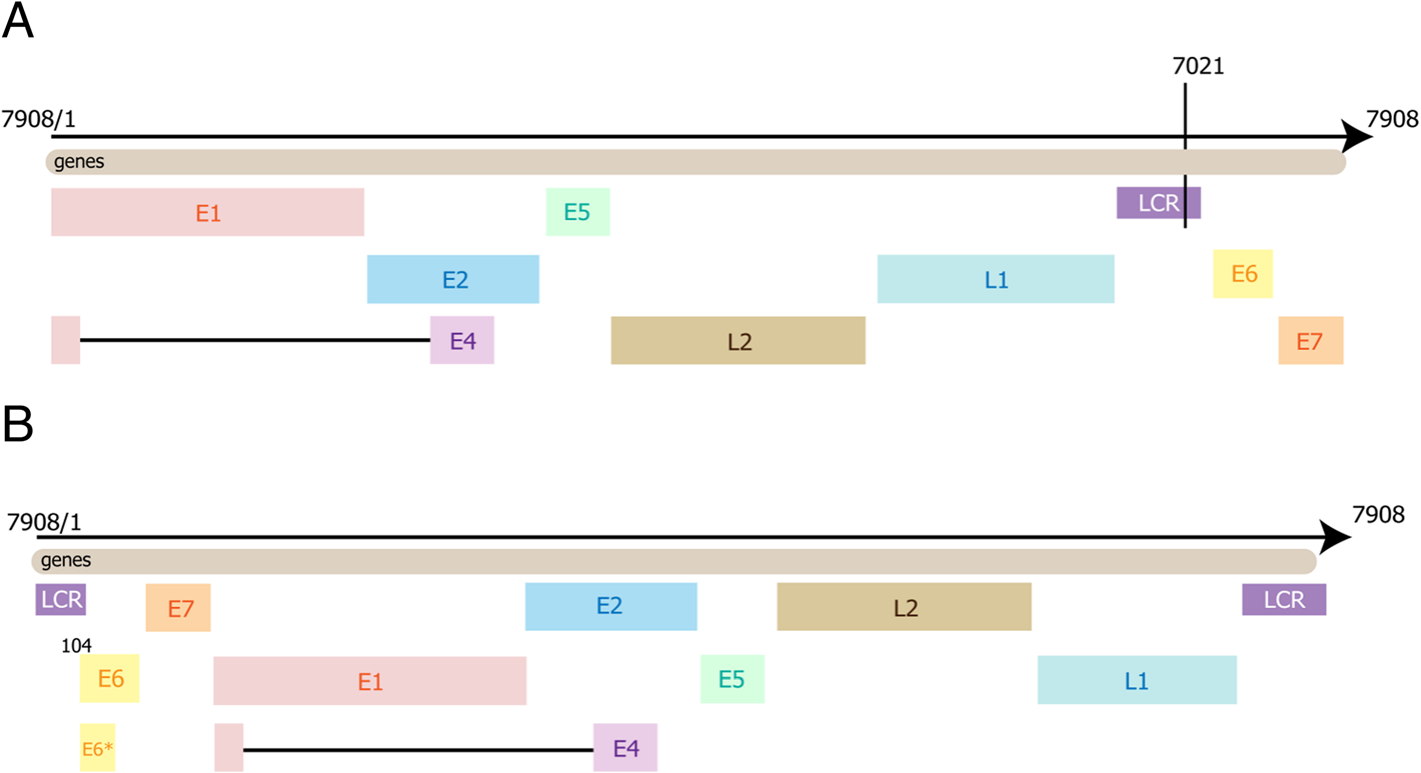
Whole genome sequence of HPV16. **a** Sequence information of HPV16 downloaded from GEO database initiated at the E1 gene site; **b** Modified sequence information of HPV16 with E6 relocated at position 104 in LCR

Human reference sequencing data were from UCSC build hg19/hg18 (http://hgdownload.cse.ucsc.edu/downloads.html#human). A file containing the entire genome sequence of HPV16, interrupted at the E1 gene initiation site (Fig. 1a), was downloaded from the GEO database. We relocated the interruption site to position 7021 of the long coding region (LCR), thus producing modified sequencing data (in Fastq format) in which E6 initiates at position 104 (Fig. 1b) [23]. HISAT2 was used to build reference files of human sequence and the modified HPV16 sequence. Meanwhile, an annotation file (in GTF format) of the modified HPV16 sequence was produced (containing E1, E2, E1^E4, E5, E6 and E7) with E6* added. Files of HPV18, HPV33 and HPV35 were similarly produced.

RNA sequencing data from18 HPV-positive HNSC patients (HPV16, *n* = 14; HPV35, *n* = 2; HPV33, *n* = 1; HPV18, *n* = 1) were downloaded from GEO [24]. The SRA Study identifier is SRP066090 (Runs: SRR2932830 to SRR2932847). RNA sequencing data of the SIHA cell line (Run: SRR1021009) and two HELA cell lines (Runs: SRR540252 and SRR629571) were also downloaded from GEO.

### Data processing

**Fig. 2:**
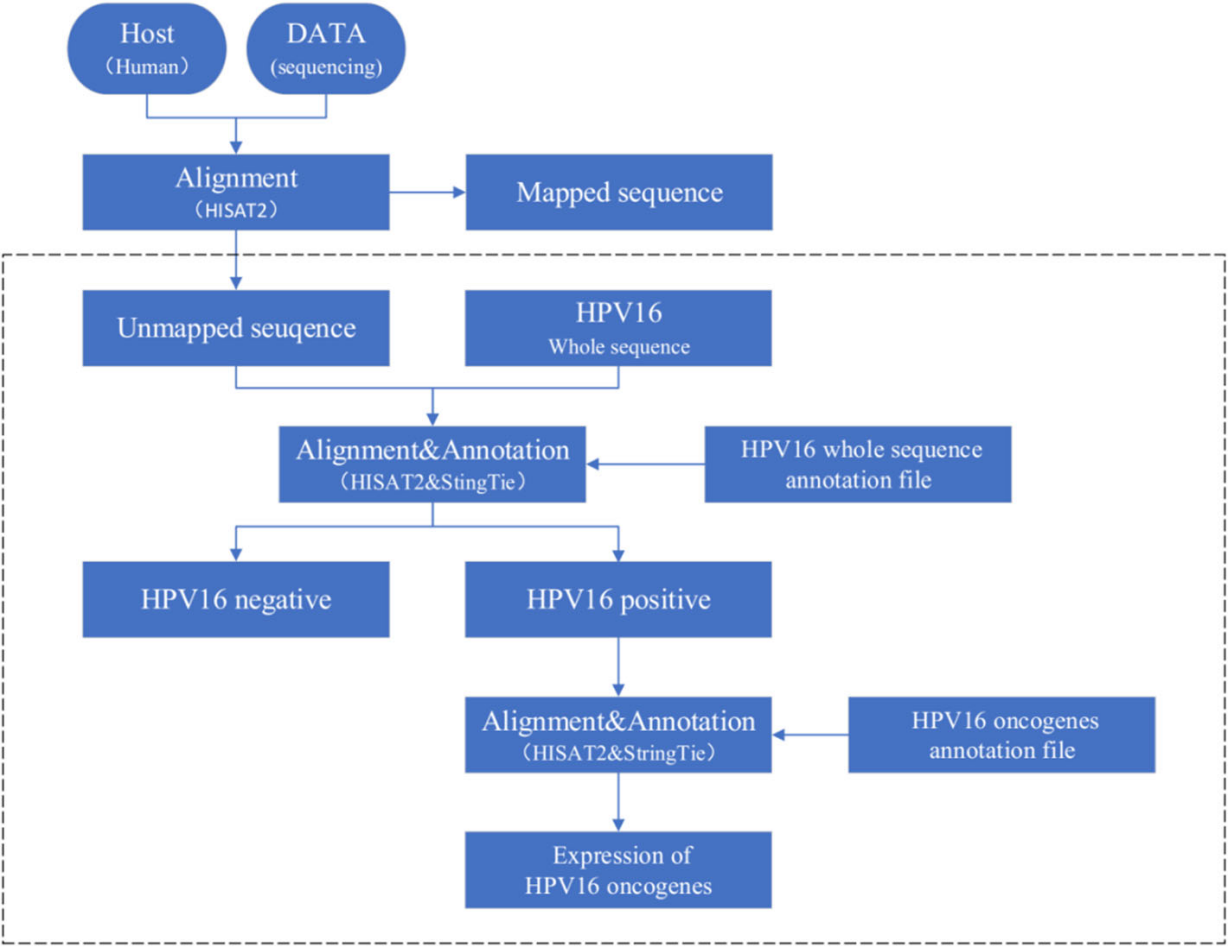
Flowchart of DisV-HPV16 Pipeline. The workflow to obtain HPV16 oncogenes expression from RNA sequencing data

DisV-HPV16 input is raw single-end or pair-end reads in Fastq format, which can be mapped to a human reference genome using alignment tool HISAT [25]. DisV-HPV16 garners all reads unmapped to the human reference genome for downstream analysis and aligns them with the entire HPV16 sequence. The results are sorted by SAM tools [26] and annotated by StringTie [27]. This step determines whether the sample is positive for HPV16. If the sample is determined to be positive for HPV16, it is annotated using the file that includes E6* (Fig. 1b). The resulting output file will contain FPKM values of HPV16 oncogenes, which can be used to estimate oncogenes expression levels (Fig. 2).

### Cell culture and RNA extraction

The head and neck cancer cell line SCC47 was kindly provided by Prof. Henning Willers (Harvard University). The cervical cancer cell line SIHA was maintained in our laboratory. Cells were grown in DMEM, 10% FBS, 100 units/mL penicillin and streptomycin at 37 °C in a humidified incubator, 5% CO_2_. For storage, cells were preserved in liquid N_2_ (− 180 °C). Total RNA (1–5 μg) obtained from SIHA and SCC47 cell cultures using Trizol was used for RT-PCR and RNA sequencing.

### RT-PCR

Total RNA (1–5 μg) was reverse transcribed into cDNA using the First strand cDNA kit (Qiagen) according to the manufacturer’s instructions. Total cDNA from HPV16-positive cell lines was amplified using Light-Cycler-FastStart DNA MasterSYBR Green I (TaKaRa Biotechnology, Dalian, China) and HPV16 primers [28]. mRNA expression was normalized using the 2^-ΔΔCt^ method based on threshold cycle value (CT value).

### RNA sequencing

After total RNA extraction and DNase I treatment, mRNA was isolated using Oligo(dT)-magnetic beads. Libraries were pair-end sequenced using Illumina HiSeqTM4000 by BGI (the Beijing Genomics Institute). The data were transferred into sequencing data via base calling, defined as raw data or raw reads and saved as FASTQ files.

### Oncogenes expression analysis

A heatmap was constructed using the pheatmap package of R. Cluster analysis was performed using R. HPV16 oncogenes expression levels were standardized by calculating the log_10_ of FPKM values.

## Results and discussion

We compared DisV-HPV16 with VirusSeq and ViromeScan, two previously available tools for the detection of viruses using data from RNA sequencing. VirusSeq required more time (2 h) than ViromeScan or DisV-HPV16 (1 h) to detect virus in RNA sequencing data from cell lines. In analyzing RNA sequencing data from the human transcriptome, the time differential was considerably greater, with VirusSeq requiring more than one day while DisV-HPV16 requiring the least time (1 h). These differences are summarized in Table 1. We ran the three software tools using the same configuration of CPU. DisV-HPV16 was overall faster in detecting virus in either cell line or human transcriptome RNA sequencing data, which we suggest is attributable to the pipeline design of DisV-HPV16 (Fig. 2).

**Table 1:** Difference in detection time of three softwares

Sample	Technology	#CPU	Detection Time		
			VirusSeq	ViromeScan	Dis-HPV16
Cellline	RNA-Seq	8	< 2 h	< 1 h	< 1 h
Human genome	RNA-Seq	8	> 1 day	< 1 h	< 1 h

Comparing with VirusSeq and ViromeScan, DisV-HPV16 needs less time under the same configuration of CPU

DisV-HPV16 detected HPV16 in SIHA cell line RNA sequencing data. Changing the DisV-HPV16 reference file allowed HPV18 detection in HELA cell line data. Furthermore, DisV-HPV16 evaluated the ratio of E2/E6 in HELA (0.007 or 0.002) and SIHA (0.24) cell line data (Table 2). Previous studies have shown that an E2/E6 ratio between 0 and 1 indicates a combined episomal plus integrated HPV16 status, while an E2/E6 ratio of 0 indicates an integrated viral status [29–31].

**Table 2:** Comparison of software accuracy and function

Cellline	HPV Genotype Detection			Ratio (E2/E6)		
	VirusSeq	ViromeScan	DisV-HPV16	VirusSeq	ViromeScan	DisV-HPV16
HELA	HPV18	HPV18	HPV18	–	–	0.007
HELA	HPV18	HPV18	HPV18	–	–	0.002
SIHA	HPV16	HPV16	HPV16	–	–	0.24

The bars denote negative results

RNA sequencing data from 18 HNSC patients were used to confirm the accuracy and sensitivity of DisV-HPV16. HPV was detected in each of 14 HPV16-positive patient samples by DisV-HPV16, as well as two samples, SRR2932838 and SRR2932841, from HPV35- and HPV33-positive patients, respectively. After changing the DisV-HPV16 reference file, we detected HPV35 in SRR2932838 and HPV33 in SRR2932841 (Table 3). DisV-HPV16 exhibited greater sensitivity than either VirusSeq or ViromeScan in detecting HPV16. We suggest that the enhanced sensitivity may be due to the new reference file we created (Fig. 1). Other genotypes of HPV were detected after changing reference files. Detection of HPV16 in HPV35-positive SRR2932838 and HPV33-positive SRR2932841 indicates the possible occurrence of co-infection in HPV-related cancers, and illustrates the sensitivity and versatility of DisV-HPV16. Previous studies have reported human co-infection by different viruses (e.g. HIV plus HPV) [32] as well as different HPV genotypes (the most frequent were HPV6, 16, 42 and 51) [33]. HIV infection is known to have a significant impact on HPV genital infection [32]. No preferential distribution of specific HPV type(s) with co-infection was identified [33]. In the future, such information may be highly useful in designing vaccination campaigns.

**Table 3:** Comparison of sensitivity in detection of HPV 16 from different samples

Subtype of HPV (n)	Sample	Software		
		VirusSeq	ViromeScan	DisV-HPV16
HPV16 (14)	SRR2932830	Over time	HPV16	HPV16
	SRR2932831	Over time	HPV16	HPV16
	SRR2932832	Over time	HPV16	HPV16
	SRR2932833	Over time	HPV16	HPV16
	SRR2932834	Over time	HPV16	HPV16
	SRR2932835	Over time	HPV16	HPV16
	SRR2932839	Over time	HPV16	HPV16
	SRR2932840	Over time	HPV16	HPV16
	SRR2932842	Over time	HPV16	HPV16
	SRR2932843	Over time	HPV16	HPV16
	SRR2932844	Over time	HPV16	HPV16
	SRR2932845	Over time	HPV16	HPV16
	SRR2932846	Over time	HPV16	HPV16
	SRR2932847	Over time	HPV16	HPV16
HPV18 (1)	SRR2932837	Over time	HPV18	HPV18
HPV33 (1)	SRR2932841	Over time	HPV33	HPV16/HPV33
HPV35 (2)	SRR2932836	Over time	HPV35	HPV35
	SRR2932838	Over time	HPV35	HPV16/HPV35

DisV-HPV16 exhibited greater sensitivity than either VirusSeq or ViromeScan in detecting HPV16

**Fig. 3:**
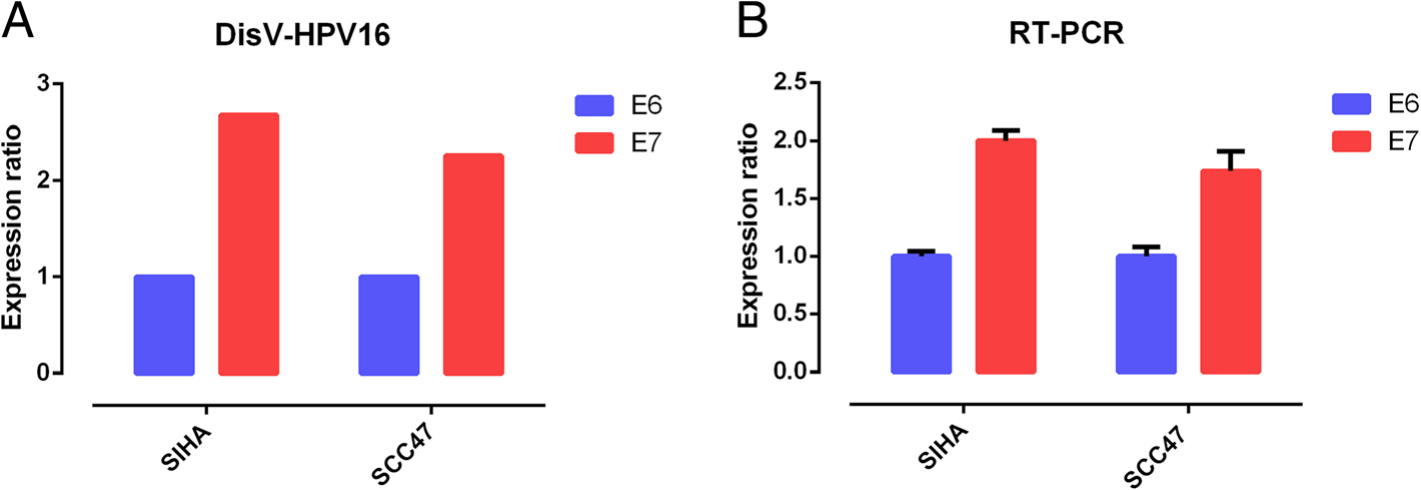
Experimental verification of DisV-HPV16 in SIHA and SCC47. **a**The ratio of E6/E7 in SIHA and SCC47 elevated by DisV-HPV16; **b** The radio of E6/E7 in SIHA and SCC47 verified by RT-PCR. The RT-PCR ratio is calculated by CT value

DisV-HPV16 accuracy was experimentally verified by RT-PCR and RNA sequencing of two HNSC cell lines, SIHA and SCC47. For SIHA cells, similar E7/E6 ratio values of 2 (average CT value: E6 = 21.27, E7 = 20.27) and 2.68 (E7 = 1,049,631.5, E6 = 391,666.94) were obtained using RT-PCR and DisV-HPV16, respectively. In SCC47 cells, RT-PCR and DisV-HPV16 analyses yielded E7/E6 values of 1.74 (average CT value: E6 = 21.73, E7 = 20.93) and 2.26 (E7 = 1,378,347.625, E6 = 610,878.63), respectively (Fig. 3). These results confirm the reliability and effectiveness of DisV-HPV16 in detecting and evaluating HPV16 oncogenes.

**Fig. 4:**
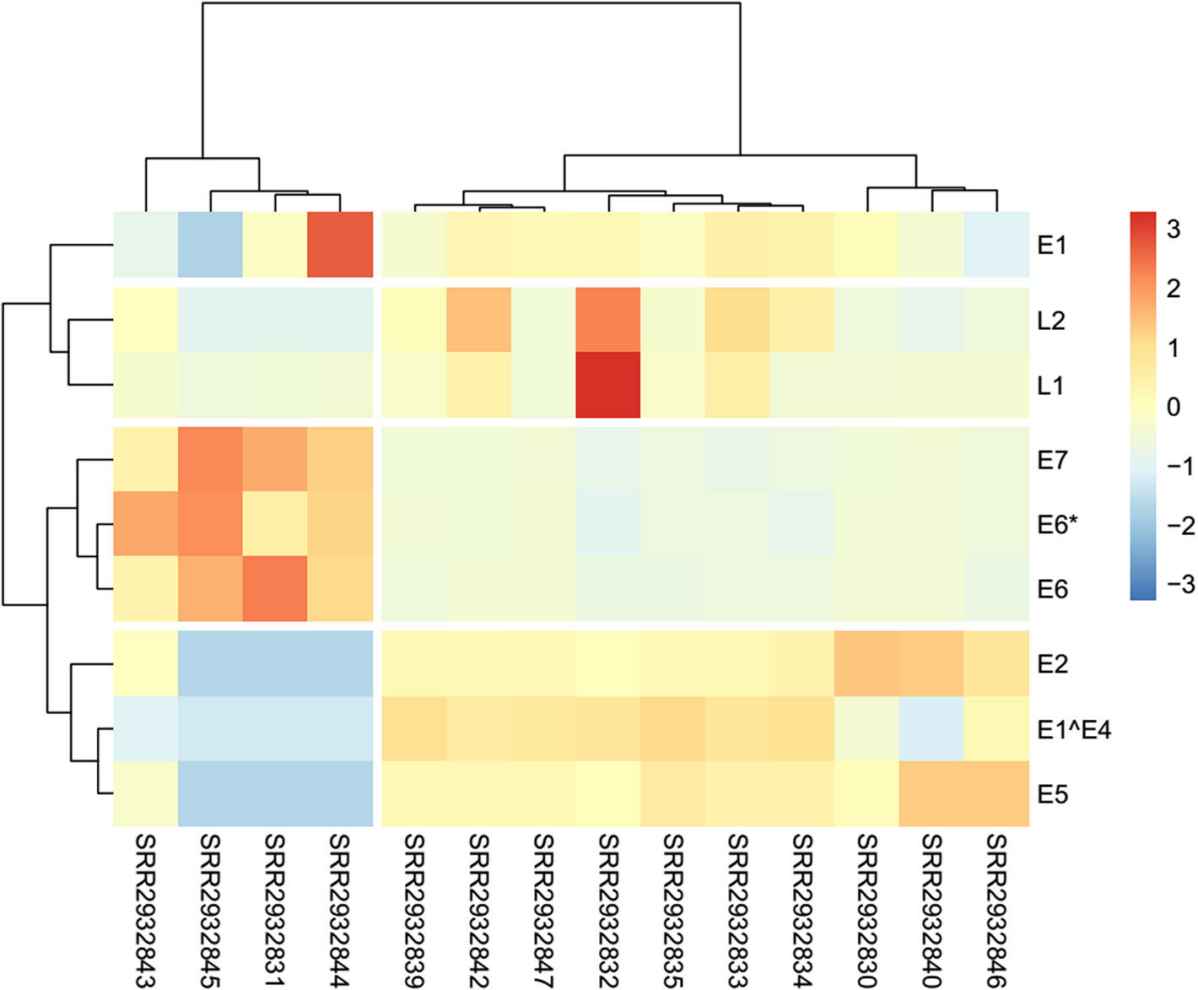
Heatmap of expression level of oncogenes in 14 HPV16 positive HNSC samples. The cell color based on the FPKM value of HPV16 oncogenes. Red means high expression while blue means low expression

DisV-HPV16 was used to evaluate HPV16 oncogenes expression in RNA sequencing data from 14 HPV16-positive patients. In a given patient sample the expression levels of different oncogenes were found to vary, as did expression of a given oncogenes among different samples. These observations are summarized in Table 4. Expression levels of HPV16 oncogenes could be clearly depicted in a heatmap based on the FPKM value for each oncogenes (Fig. 4). The 14 samples segment into two groups **(**four on the left and ten on the right in Fig. 4). Three early genes E6, E7 and E6* are in one cluster while E2, E5 and E1^E4 are in the other, indicating opposite oncogenes expression trends in the two clusters. This result might reflect E2-induced increase in viral replication via splicing-related activities [34], which results in high-level expression of E6 and E7. This may result in clinically significant differences among individual patients suffering from HPV16-positive head and neck cancers.

**Table 4:** Expression levels of HPV16 oncogenes in 14 HPV16-positive samples

Samples	E1	E1^E4	E2	E5	L2	L1	E6*	E6	E7
SRR2932830	4.69	5.52	5.55	5.78	3.11	3.44	5.31	5.26	5.66
SRR2932831	4.65	0.00	0.00	3.17	2.21	2.96	5.71	5.82	6.23
SRR2932832	4.72	5.90	5.30	5.76	4.02	4.90	4.61	5.11	5.46
SRR2932833	4.78	5.89	5.35	5.85	3.82	4.36	5.18	5.14	5.51
SRR2932834	4.75	5.93	5.37	5.85	3.67	3.49	4.91	5.17	5.58
SRR2932835	4.64	5.96	5.34	5.90	3.30	3.81	5.13	5.11	5.59
SRR2932839	4.59	5.94	5.35	5.81	3.52	3.81	5.31	5.19	5.68
SRR2932840	4.57	4.58	5.54	6.00	2.61	3.54	5.31	5.26	5.69
SRR2932842	4.74	5.85	5.33	5.81	3.90	4.31	5.28	5.27	5.64
SRR2932843	4.40	4.94	5.28	5.68	3.48	3.75	5.95	5.50	5.99
SRR2932844	5.08	0.00	3.01	3.15	0.00	3.32	5.85	5.66	6.16
SRR2932845	3.21	0.00	2.93	0.00	0.00	0.00	6.00	5.73	6.29
SRR2932846	4.31	5.76	5.46	6.01	3.06	3.61	5.22	5.11	5.62
SRR2932847	4.71	5.88	5.34	5.81	3.15	3.18	5.37	5.27	5.70

Note: Standardized numerical value represented expression levels of HPV16 oncogenes

## Conclusions

The new DisV-HPV16 software not only detected the existence of distinct HPV genotypes (HPV16, HPV18, HPV33 and HPV35) in RNA sequencing data (simply by changing reference files) but also revealed the expression levels of HPV16 oncogenes. DisV-HPV16 provides enhanced virus detection and analysis capabilities based on RNA sequencing data and also enlarges the potential for understanding the effects of viral genes on the host genome and elucidating key features of the virus-host relationship. In the present study we tested DisV-HPV16 on four HPV genotypes. Whether the software can be of value for detection and evaluation of additional viruses will be determined in future studies.

### Availability and requirements

Project name: DisV-HPV16.

Project home page: https://github.com/ybq1204/DisV-HPV16

Operating system(s): Linux.

Programming language: Shell.

Other requirements: None.

License: None.

Any restrictions to use by non-academics: None.

## Data Availability

Head and neck cancer sequencing data are downloaded from NCBI database (SRP066090, SRR1021009, SRR540252 and SRR629571). SCC47 was kindly provided by Prof. Henning Willers (Harvard University). The cervical cancer cell line SIHA was maintained in our laboratory. And the sequencing data of cell lines are available.

## Abbreviations

CC: Cervical carcinoma
DisV-HPV16: Discover Virus of HPV16
HNSC: Head and neck squamous carcinoma
HPV: Human papillomavirus
